# Lasing in silicon–organic hybrid waveguides

**DOI:** 10.1038/ncomms10864

**Published:** 2016-03-07

**Authors:** Dietmar Korn, Matthias Lauermann, Sebastian Koeber, Patrick Appel, Luca Alloatti, Robert Palmer, Pieter Dumon, Wolfgang Freude, Juerg Leuthold, Christian Koos

**Affiliations:** 1 Institute of Photonics and Quantum Electronics (IPQ), Karlsruhe Institute of Technology (KIT), 76131 Karlsruhe, Germany; 2 Institute of Microstructure Technology (IMT), Karlsruhe Institute of Technology (KIT), 76344 Eggenstein-Leopoldshafen, Germany; 3 Department of Information Technology, IMEC, 9000 Gent, Belgium

## Abstract

Silicon photonics enables large-scale photonic–electronic integration by leveraging highly developed fabrication processes from the microelectronics industry. However, while a rich portfolio of devices has already been demonstrated on the silicon platform, on-chip light sources still remain a key challenge since the indirect bandgap of the material inhibits efficient photon emission and thus impedes lasing. Here we demonstrate a class of infrared lasers that can be fabricated on the silicon-on-insulator (SOI) integration platform. The lasers are based on the silicon–organic hybrid (SOH) integration concept and combine nanophotonic SOI waveguides with dye-doped organic cladding materials that provide optical gain. We demonstrate pulsed room-temperature lasing with on-chip peak output powers of up to 1.1 W at a wavelength of 1,310 nm. The SOH approach enables efficient mass-production of silicon photonic light sources emitting in the near infrared and offers the possibility of tuning the emission wavelength over a wide range by proper choice of dye materials and resonator geometry.

## Abstract (web summary)

On-chip light sources for silicon photonic circuits remain a challenge since the indirect bandgap of silicon prevents efficient light emission. The authors demonstrate that lasing can be achieved by combining standard silicon-on-insulator waveguides with dye-doped organic cladding materials to provide optical gain.

## Graphical abstract

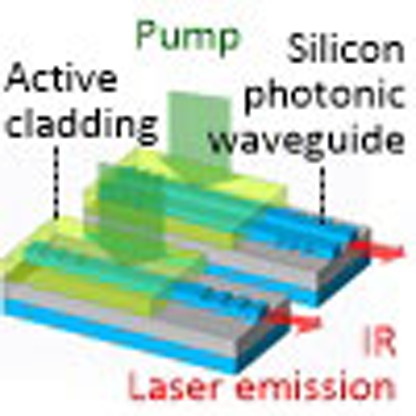

Silicon photonics allows fabrication of nanophotonic devices using commercial CMOS facilities and is therefore a highly attractive platform for large-scale photonic integration^1^,^2^. However, while a wide variety of silicon-based optical and electro-optical devices has been demonstrated over the last years^3^, efficient on-chip light sources still represent a challenge^4^ due to the indirect bandgap of silicon. Previously reported all-silicon light sources rely on stimulated Raman scattering as a gain mechanism^5^,^6^,^7^,^8^. One drawback of these schemes is that they require coupling of external pump lasers to single-mode on-chip waveguides. In early demonstrations, Raman laser cavities were formed by incorporating nanophotonic silicon waveguides into fibre-based off-chip laser cavities to enable synchronization of the cavity round-trip time with a pulsed pump^5^,^6^. These devices cannot be miniaturized. In contrast to that, silicon photonic Raman lasers with on-chip cavities are compact and enable continuous-wave (CW) lasing^7^,^8^, but require either strong pump lasers in combination with reverse-biased p-i-n-junctions or provide only limited output power in the microwatt range. Hybrid approaches, in which silicon is combined with direct-bandgap III–V compound semiconductors, allow for electrically pumped amplifiers^9^ and lasers^10^,^11^, but fabrication requires sophisticated and technologically challenging die-to-wafer bonding processes or advanced technology for the direct growth of III–V quantum dots^12^ on silicon. Regarding monolithic integration of light sources on silicon, an electrically pumped CW germanium-on-silicon laser has been demonstrated by using a combination of tensile strain and n-doping of the germanium to enable direct-bandgap transitions in thin germanium layers that are grown on silicon substrates^13^. More recently, lasing has been shown without introducing mechanical strain by using a germanium–tin alloy^14^ on silicon. However, fabrication of such devices requires advanced crystal growth techniques and technologically challenging fabrication processes. As an alternative, combinations of erbium-doped active cladding materials and SOI waveguides have been proposed^15^,^16^ and experimentally investigated^17^,^18^. However, erbium features a rather small emission cross section and hence small gain. As a consequence, lasing in integrated erbium-clad devices has so far only been demonstrated for low-loss silicon nitride waveguides^19^, but not for high-index-contrast SOI waveguides. Regarding peak output power, even the most outstanding on-chip silicon-based lasers are currently limited to ∼100 mW or less^12^,^20^,^21^.

In this work, we demonstrate that lasing can be achieved by combining standard silicon-on-insulator (SOI) waveguides with dye-doped organic cladding materials. This concept of silicon–organic hybrid (SOH) integration is particularly well-suited for flexible and low-cost mass-production of silicon photonic light sources emitting in the near infrared. In a proof-of-principle experiment, we demonstrate pulsed lasing at room temperature with peak output powers of up to 1.1 W at a wavelength of 1,310 nm. Gain is provided by a near-infrared dye that was previously demonstrated to enable lasing in plastic waveguides^22^. More general, exploiting the virtually unlimited variety of organic optical cladding materials, SOH integration allows to complement silicon photonics with novel functionalities while still preserving the strengths of highly standardized CMOS processing^23^. Our proof-of-principle demonstration of SOH light sources complements recent work on SOH integration, comprising high-speed all-optical signal processing^24^, broadband electro-optic modulators^25^,^26^ and highly efficient low-power phase shifters^27^.

## Results

### Concept and fabrication of SOH lasers

**Figure 1:**
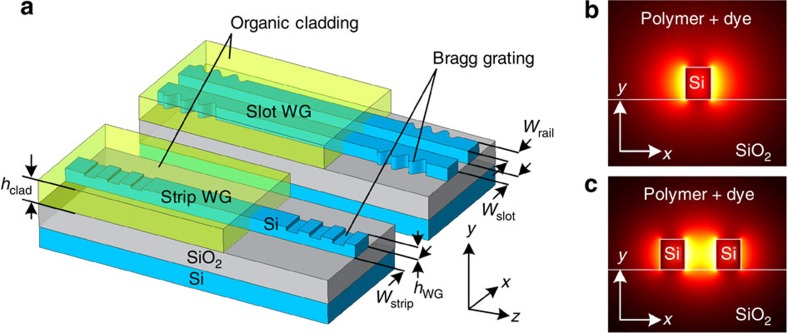
SOH laser concept. (**a**) Light is guided by SOI strip or slot waveguides consisting of thin silicon nanowires (width *w*_strip_≈150–500 nm, height *h*_WG_≈200–350 nm) that are optically isolated from the silicon substrate by a thick oxide (*h*_SiO2_≈2 μm). Optical gain is provided by a fluorescent organic cladding material (thickness *h*_clad_≈500±50 nm), which entirely covers the strip or fills the slot (*w*_rail_≈100–200 nm, *w*_slot_≈50–200 nm). The optical pump is either launched from above or injected into the waveguide at one of the facets. Bragg reflectors can be used to provide wavelength-selective optical feedback. Interaction of the guided light with the active cladding is maximized by the design of the waveguides. (**b**) Dominant electric field component (*E*_x_) of the fundamental quasi-TE mode for a narrow strip waveguide (colour coding: lighter colours for higher magnitude). A large fraction of the guided mode propagates in the cladding. (**c**) Dominant electric field component (*E*_x_) of the fundamental quasi-TE mode for a slot waveguide consisting of two tightly spaced silicon rails. Discontinuities of the dominant horizontal electric field component lead to a strong field enhancement within the slot region and hence to a strong interaction with the active cladding.

The basic idea of an SOH laser is illustrated in Fig. 1a. The devices consist of SOI waveguides, which are terminated at both ends with Bragg reflectors^28^ and which are covered by a fluorescent organic cladding material suitable for stimulated emission when optically pumped. For efficient light emission, the interaction of the guided optical mode with the active cladding must be maximized. This can be accomplished by using a narrow silicon strip waveguide, for which a large fraction of the guided mode reaches into the cladding, Fig. 1b. Alternatively, a slot waveguide can be used, which consists of two closely spaced silicon rails, Fig. 1c. In both cases, the dominant horizontal electric field component (*E*_*x*_) of the optical quasi-TE mode experiences strong field discontinuities at the high-index-contrast sidewalls. For the slot waveguide, this leads to an especially pronounced field enhancement within the slot^29^, and hence to a strong interaction with the active cladding.

**Figure 2:**
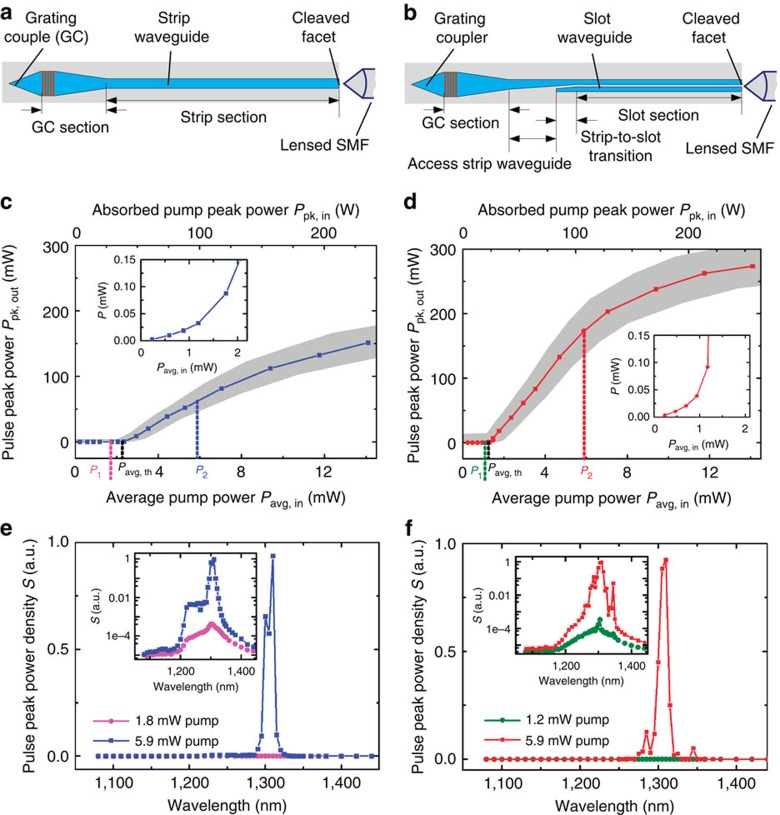
Experimental proof of lasing in SOH strip and slot waveguides. The cladding consists of the commercially available dye IR26 (ref. 22) dispersed in a PMMA matrix. Cavity mirrors are formed by one cleaved waveguide facet and a GC. The GC is designed for coupling 1,550 nm light from an optical fibre to the strip and exhibits substantial back-reflection at the laser emission wavelength of 1,310 nm. For both the strip and the slot waveguide, the cavities are ∼4.8 mm long. The laser output power is measured in a lensed SMF that collects light from the waveguide facet. (**a**) Strip waveguide consisting of a 450 μm long GC section and a 4.3 mm long strip section (waveguide height *h*_WG_≈220 nm, width *w*_strip_≈210 nm). (**b**) Slot waveguide comprising a 450 μm long GC section, a 235 μm long access strip waveguide, a 300 μm long strip-to-slot transition, and a 3.8 mm long slot waveguide section (rail width *w*_rail_≈180 nm, slot width *w*_slot_≈215 nm). (**c**) Peak output power *P*_pk, out_ (all polarizations) in lensed SMF versus illuminating average pump power *P*_avg, in_ for the strip-waveguide cavity. A clear pump power threshold of *P*_avg, th_=2.3 mW can be observed. The measured incident average pump power (bottom scale) *P*_avg, in_ is used to calculate the absorbed pulse peak power (top scale) *P*_pk,in_, taking into account the specific parameters of the waveguide, see the section ‘Estimation of pump power levels' of the Methods and Supplementary Note 1. The grey-shaded area indicates an estimate of the accuracy of the measurement. For the uncertainty of the pump power, we use a relative standard error of ±14%, see the section ‘Estimation of emission power levels' of the Methods for a more detailed explanation. Regarding the uncertainty of the emitted power, we estimate a relative standard error of ±10% for all pump powers of *P*_p1_=5 mW or more. Below pump powers of *P*_p1_=5 mW, we assume a constant absolute error which corresponds to the ±10% relative standard error at *P*_p1_=5 mW. Note that the grey ranges correspond to a coarse, but conservative estimate of the measurement uncertainties. *P*_1_ and *P*_2_ denote the pump powers for which the spectra in **e** are recorded. (**d**) Peak output power *P*_pk, out_ in SMF versus incident average pump power *P*_avg, in_ for the slot-waveguide cavity. The grey-shaded areas indicate again an estimate of the accuracy of the measurements, see the section ‘Estimation of emission power levels' of the Methods. A threshold pump power *P*_avg, th_=1.3 mW is found, which corresponds to 60% of the threshold for the strip waveguide. *P*_1_ and *P*_2_ denote the pump powers for which the spectra in **f** are recorded. (**e**) Emission spectra below (ASE, magenta) and above threshold (blue) for the strip-waveguide cavity. The spectrum is given in arbitrary units of the spectral pulse peak power density *S* recorded with a resolution bandwidth of 5 nm (inset with logarithmic scale). Above threshold, the emission spectrum narrows considerably. (**f**) Emission spectra below (ASE, green) and above threshold (red) for the slot-waveguide cavity (inset with logarithmic scale). Also here, the emission spectrum narrows considerably above threshold. For all spectra, the resolution bandwidth amounts to 5 nm to allow for detection of weak ASE and strong laser emission with the same measurement system. High-resolution spectra above lasing threshold have been taken at smaller RBW of 0.2 and 0.05 nm, see Supplementary Note 2 and Supplementary Fig. 3.

We prove the viability of the concept by investigating a simple test structure. To this end, strip and slot waveguides of 4.8-mm length were fabricated using a state-of-the-art SOI CMOS-based process^30^. The waveguides are embedded into a solid active cladding consisting of a poly(methyl methacrylate; PMMA) matrix doped with 1 wt% of the commercially available dye IR26 (refs 22, 31) having a maximum fluorescence at 1,150 nm, see Supplementary Fig. 1. The cladding is deposited in a single post-processing step using standard spin-coating techniques. Scanning electron microscope (SEM) images of coated and uncoated samples can be found in Supplementary Fig. 2, showing that the PMMA cladding fills the slot completely without forming any voids. To enable laser operation in a wide wavelength range, we omit the wavelength-selective Bragg reflectors shown in Fig. 1 and exploit spurious back-reflection from cleaved waveguide facets and from on-chip grating coupler (GC) structures, see Fig. 2a,b. For the cleaved facets, power reflection factors between 4 and 8% are estimated. Light emission from the cleaved facets is coupled to lensed standard single-mode fibres (SMF). For coarse alignment of the fibres, we use 1,550-nm light coupled to the SOH waveguide via the GC. The GC is optimized for operation at a wavelength of 1,550 nm and exhibit spurious back-reflection when operated at the laser emission wavelength of 1,310 nm. This reflection amounts to a few per cent and is comparable to that of the cleaved facet. A more detailed description of device fabrication can be found in the section ‘Fabrication of SOH lasers' of the Methods.

Lasing could be demonstrated despite the comparatively low quality of the Fabry–Perot laser resonator, underlining the high potential of using dye-doped active claddings as gain media. In the experiment, the devices are pumped from above by a free-space line-focus beam using a pulsed laser with a wavelength of 1,064 nm, a pump pulse duration of 0.9 ns (full width at half maximum, FWHM), and a pulse energy of up to 1.2 mJ at a repetition rate of 13.7 Hz. The duration of the emitted laser pulses amounts to ∼0.6 ns. Note that this is much longer than the cavity round-trip time of the laser, and pulsed operation is caused solely by the fact that the pump source is switched on only for certain time intervals. The effective lifetime of the excited state amounts to ∼10 ps and is much shorter than the durations of the pump and the emission pulses. We may hence assume that the lasing process is close to its steady-state. The experimental setup is explained in more detail in the section ‘Experimental demonstration of laser emission' of the Methods, which is followed by an estimation of the pump and emission power levels.

### Characterization and experimental proof of lasing

We measured the laser output power in the SMF as a function of the pump power for both the strip and the slot waveguide, Fig. 2c,d. In both cases, a clear threshold can be observed at a launched average pump power of approximately 2.3 mW for the strip, and approximately 1.3 mW for the slot waveguide. The absorbed peak power at threshold in the vicinity of the waveguide can be roughly estimated to be 38 W for the strip, and 24 W for the slot waveguide, taking into account specific parameters of the individual waveguides, see the sections ‘Experimental demonstration of laser emission' and ‘Estimation of emission power levels' of the Methods and Supplementary Note 1 for more details.

The existence of the threshold indicates laser emission. The measured threshold level is in reasonable agreement with theory, see the section ‘Consistence of resonator characteristics and threshold pump power' of the Methods for a more detailed discussion. To rule out any laser look-alikes, we investigate further criteria formulated by Samuel *et al.*^32^ Below threshold, only amplified spontaneous emission is to be seen, which increases exponentially with the pump power, see insets of Fig. 2c,d. Above threshold, the output power increases linearly with the pump power. For very high pump powers, the laser power saturates. The saturation is attributed to absorption bleaching at the pump wavelength and to pump-induced free-carrier absorption (FCA) in the SOI waveguide, see the section ‘Optically induced losses and dynamical behaviour' of the Methods for a more detailed discussion.

Moreover, we investigate the emission spectra from the strip and slot waveguides below and above threshold, see Fig. 2e,f. Broadband amplified spontaneous emission can be observed for operation below threshold, see insets of Fig. 2e,f (logarithmic scale). When pumped above threshold, the emission spectrum narrows considerably. In Fig. 2e and f, the observed linewidth appears slightly larger than the resolution bandwidth of the spectrometer (RBW=5 nm). We attribute this to a multitude of different longitudinal cavity modes which oscillate simultaneously at every pump pulse, see Supplementary Note 2 and Supplementary Fig. 3 for a more detailed description.

Above threshold, the optical output of slot waveguides and of narrow strip waveguides is laterally single-mode, which can be inferred from the observation that there is a single well-defined optimum spot when coupling to a lensed SMF. For strip waveguides, lasing in higher-order lateral modes can be observed for waveguide widths of ∼300 nm or more as reported in more detail in the next section ‘Influence of waveguide geometry'. For the devices shown in Fig. 2, the emitted light is predominantly polarized in the horizontal direction as is expected for lasing of the quasi-TE mode. The polarization extinction ratio (ER) is about 8 dB for both devices in Fig. 2. To confirm that the dye is indeed responsible for lasing, we prepared reference samples without dye in the PMMA cladding. These samples do not show noticeable light emission. Moreover, without the silicon waveguide but with dye in the cladding, only spontaneous emission is observed. These findings exclude any laser look-alikes and confirm the working principle of the SOH laser concept.

### Influence of waveguide geometry

**Figure 3:**
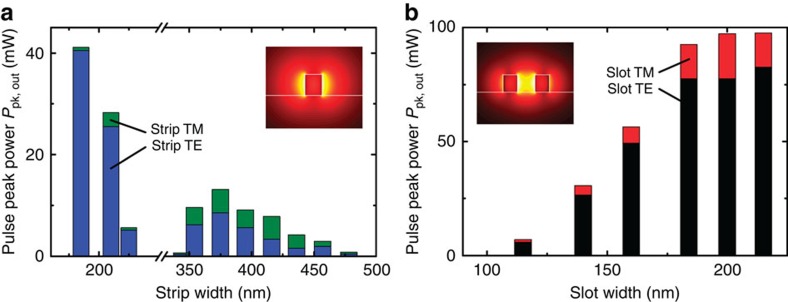
Geometry-dependent peak output power *P*_pk, out_ coupled into a lensed SMF for strip and slot waveguides. The resonator relies on back-reflection from one cleaved waveguide facet and from a GC operated far from its design wavelength of 1,550 nm. Quasi-TE and quasi-TM polarizations are measured separately. The average incident pump power is 5 mW for all samples. In the bar diagram, the differently coloured areas represent the contributions of the quasi-TE and the quasi-TM polarization to the total output power; the total bar height corresponds to the total emission. Insets: Dominant electric field magnitudes of the fundamental quasi-TE modes. (**a**) Strip-waveguide cavity. The laser power is largest when the strip width is smallest such that the guided light extends far into the cladding. The secondary maximum at 375 nm is due to lasing of the next higher-order mode (quasi-TE_10_), which also has a strong overlap with the active cladding, but is not guided for smaller strip widths. (**b**) Slot-waveguide cavity. An increase of the slot width leads to an increase of the field confinement in the cladding and to an expansion of the region in which the active dye interacts with the optical mode. As a consequence, the lasing power increases with slot width. For large slot widths, the fundamental mode is only weakly guided, and the laser power does not increase further. The rail width has only a minor influence (not shown) and is fixed at 170 nm.

Regarding the influence of waveguide geometry on the performance of the SOH lasers, we find that lasing with high output powers can be achieved with a wide range of waveguide dimensions and that the output power is clearly related to the overlap of the guided mode with the active cladding. The geometry-dependent output power levels of different waveguide geometries are shown in Fig. 3. For the strip waveguide, we vary the width, Fig. 3a, whereas for the slot waveguide, the rail width is fixed to 170 nm and the slot width is varied, Fig. 3b. The length of the active section amounts to 4 mm for all devices. As before, the resonator is formed by back-reflection from a cleaved waveguide facet and from a GC operated far from its design wavelength of 1,550 nm. The experimental setup is the same as before and described in the section ‘Experimental demonstration of laser emission' of the Methods. The average pump power is fixed to 5 mW. The coloured areas of each bar in Fig. 3 represent the respective contributions of quasi-TE (blue) and quasi-TM polarization (green) to the total output power. For the strip waveguide, the laser power is largest when the strip width is smallest, that is, when the mode fields extend far into the active cladding. The second maximum at *w*_strip_=375 nm is due to lasing not only of the fundamental mode, but also of the next higher-order quasi-TE_10_ mode, which also strongly interacts with the cladding. The polarization ER reaches a maximum of (18±2 dB) for the narrowest strip waveguides we investigated.

In Fig. 3b, we consider slot waveguides and vary the slot width while keeping the rails widths at a constant value of 170 nm. The TE mode dominates laser emission, since interaction with the cladding is enhanced by the electric field discontinuities at the high-index-contrast sidewalls of the slot, as can be seen by comparing the field interaction factors^33^ Γ_clad, TE_ and Γ_clad, TM_ for the two polarizations, see Supplementary Table 1 and Supplementary Note 1. Moreover, the emitted laser power increases with slot width. This is to be expected since larger slot widths lead to both larger field interaction factors of the guided mode with the active cladding and to a larger volume in which dye molecules can interact with the guided mode. For very large slot widths, the slot mode is only weakly guided and leaks into the high-index silicon substrate. As a consequence, the output power does not increase further. The polarization ER remains nearly constant and reaches a maximum of 8±2 dB for a slot width of 140 nm. For wider and narrower slots, the ER is slightly smaller.

The optimum choice of the waveguide geometry depends on the desired balance between output power and polarization ER: High power output and a moderate ER when using slot waveguides have to be compared with about half the output power and a high polarization ER obtained from narrow strip waveguides. Using state-of-the-art CMOS fabrication, waveguide dimensions can be reproduced with tolerances of significantly less than 10 nm, which does not influence output power or polarization ER of the SOH lasers to a significant degree. SOH device performance can hence be expected to be resilient against fabrication inaccuracies.

### Dynamic emission behaviour

**Figure 4:**
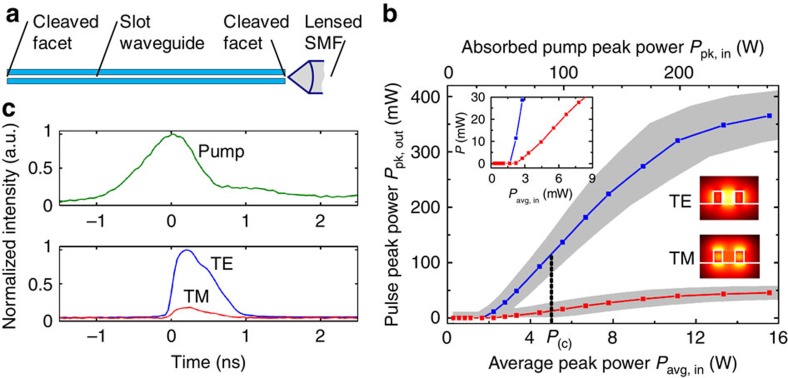
Lasing in a SOH slot waveguide. In this experiment, cavity mirrors are formed by cleaved waveguide facets on both ends. The cavity length is 3.8 mm, the waveguide height amounts to 220 nm, and for the rail and the slot width, values of *w*_rail_=(160±15) nm and *w*_slot_=(180±15) nm were extracted from scanning electron microscope (SEM) images. (**a**) Schematic top view of the slot waveguide. (**b**) Peak output power in the lensed SMF for quasi-TE and quasi-TM mode versus incident average pump power. The absorbed pump peak power is estimated from the measured incident average pump power, see the section ‘Estimation of pump power levels' of the Methods. The grey-shaded areas indicate an estimate of the accuracy of the measurement. For the uncertainty of the pump power, we assume a ±14% relative standard error. Regarding the uncertainty of the emitted power, we estimate a relative standard error of ±10% for all pump powers of *P*_p1_=5 mW or more. Below pump powers of *P*_p1_=5 mW, we assume a constant absolute error which corresponds to the ±10% relative standard error at *P*_p1_=5 mW. More details can be found in the section ‘Estimation of emission power levels' of the Methods. Note that the grey ranges correspond to a coarse, but conservative estimate of the measurement uncertainties. Inset: zoom-in of pulse peak power at low pump powers, demonstrating sharp thresholds for both TE and TM mode. (**c**) Temporal shape of the pump pulse at an average power of 5 mW (green) and of the corresponding emission pulses (TE, blue; TM, red). The shape of the pump pulse was measured by averaging over 16 pulses and normalizing to a peak value of 1. Likewise, the emission pulses were measured in the SMF and averaged over 16 pulses. In the plot, the peak of the TE emission has been normalized to 1, and the TM emission is plotted at the same scale. The exact delay between pump and emission cannot be exactly determined due to modal and chromatic dispersion in the standard SMF. The peak pump power was determined with a relative standard error of ±14%; for the peak power of the emitted pulse the relative standard error is ±10%, see the sections ‘Estimation of emission power levels' and ‘Estimation of pump power levels' of the Methods for a more detailed discussion.

The achievable peak output power of the SOH lasers is remarkable: For an SOH slot waveguide with cleaved facets on both sides, we measured peak output powers of up to 365 mW in the attached SMF, see Fig. 4a,b. The fibre–chip coupling losses are estimated to be (5±1) dB, which leads to peak powers of (30.3±1.0) dBm at the output facet, that is, 1.1 W that could be coupled to an on-chip nanophotonic SOI waveguide. This is the highest peak power emitted from a silicon-based laser with on-chip cavity so far. A more detailed discussion can be found in the section ‘Estimation of emission power levels' of the Methods.

The time-dependent emission of the slot waveguide laser is depicted in Fig. 4c for both polarizations, recorded at an average pump power of 5 mW. We observe laser emission into both the quasi-TE and quasi-TM mode, which we attribute to local gain depletion: for large slot widths, the TE and TM modes occupy different cross-sectional domains of the active cladding, see insets in Fig. 4b, and lasing may therefore occur simultaneously in both polarizations. Since the overlap of the quasi-TE slot mode with the active cladding is larger than that of the TM mode, the TE mode experiences higher gain and hence dominates lasing with a polarization ER of 9 dB. The TE and TM emission spectra are similar—see Supplementary Fig. 3 and Supplementary Note 2 for a more detailed discussion.

Regarding the pulse shapes, we find that the mean FWHM duration of emission amounts to 0.6 ns, which is shorter than the pump pulse FWHM of 0.9 ns. Moreover, the emission pulse features an asymmetric shape and is delayed with respect to the pump pulse. The delay is attributed to the fact that laser emission can only set in once the pump intensity exceeds the threshold level. Note that the relative timing of pump pulse and emission pulses is subject to uncertainties of approximately ±100 ps due to different propagation delays in the fibre-based measurement setup, see the section ‘Optically induced losses and dynamical behaviour' of the Methods for more details. The instantaneous pump power at the onset of laser emission can therefore not be directly associated with the threshold pump power level identified in Fig. 4b. The asymmetric shape of the emission pulse might be caused by nonlinear absorption and subsequent relaxation processes in the active cladding. This aspect requires further investigation.

## Discussion

SOH lasers have the potential to cover a broad range of different emission wavelengths between 1.1 and 1.6 μm by using suitable dye materials^34^,^35^. Due to the high output power, the devices may even be used for exploiting nonlinear optic effects in nanophotonic waveguides. The SOH lasers are remarkably robust: during our experiments, we did not observe significant degradation of the devices, even though they were tested repeatedly over several weeks without taking any specific efforts with respect to encapsulation. This first indication of high stability of the SOH lasers is in good agreement with previous observations, which have shown that photo bleaching of IR26 can be neglected at our pump wavelength of 1,064 nm (ref. 22). A detailed investigation of the stability of SOH lasers is subject of further research.

The devices presented in this paper are first-generation prototypes with considerable room for improvement. In particular, lasing threshold and linewidth of optical emission can be reduced by using optimized Bragg reflectors or ring resonators for optical feedback. Moreover, according to our study of the laser dynamics, we expect that better efficiency and lower threshold can be achieved by avoiding FCA as an important loss mechanism of the cavity. To this end, one might consider dyes that allow for pump wavelengths above the absorption edge of silicon^35^. Moreover, the pump efficiency can be improved considerably by guiding the pump light along the SOI waveguide to concentrate it in the active zone. This could be achieved by using an additional polymer waveguide around the SOI waveguide. High duty cycles or CW emission are in general difficult to achieve in dye lasers due to triplet-state excitation and subsequent photo-induced degeneration. This deficiency could be overcome by doping the matrix material with triplet-state quenching or triplet-trapping species of molecules^36^, by using optofluidic concepts^37^ or by choosing other gain materials such as lanthanide ions or colloidal quantum dots^38^,^39^ that might even be suited for direct electrical pumping^40^.

Nevertheless, even without CW operation, SOH lasers enable greatly simplified one-step fabrication processes for realizing thousands of light sources directly integrated into silicon photonic circuitry. Such light sources lend themselves to a wide range of applications such as biosensing^41^, where pulsed operation with low-duty cycles is sufficient, where cost-efficient mass fabrication is essential to enable disposable chips for one-time use, and where pump efficiency is secondary. Moreover, the high peak power of the SOH lasers might open interesting opportunities in nonlinear infrared spectroscopy. Further investigation of the dynamics, optimization of the active cladding, and the use of better resonators should help enlarging the application range. We therefore believe that the present approach will be the basis for a novel class of silicon photonic on-chip sources that stand out due to their high peak output power and ease of fabrication.

## Methods

### Fabrication of SOH lasers

Waveguides were fabricated on SOI wafers from SOITEC using a CMOS pilot line based on 193-nm deep-ultra-violet lithography^30^. All waveguides have a height of *h*_WG_=220 nm and are optically isolated from the silicon substrate by a buried oxide (SiO_2_) layer of thickness *h*_SiO2_=2 μm.

The gain medium is deposited on the silicon waveguides in a single post-processing step by spin-coating. The active organic cladding consists of a PMMA matrix which is doped with 1 wt% of the commercially available dye IR26 (ref. 22). The final thickness of the cladding amounts to *h*_clad_≈(500±50) nm. The measured absorption and fluorescence spectra of a liquid dye solution are depcited^31^ in Supplementary Fig. 1, exhibiting a fluorescence emission peak at 1,130 nm. When using the dyes in an extended waveguide structure, the emission peak of IR26 shifts to ∼1,300 nm due to self-absorption along the waveguide in the overlap region of the emission and the absorption spectra^42^. This is in good agreement with the laser emission wavelength observed in ref. 22.

### Experimental demonstration of laser emission

**Figure 5:**
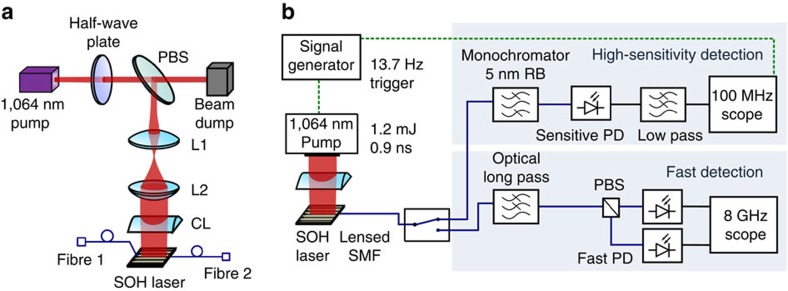
Measurement setup. (**a**) Pump light at 1,064 nm is focused on the SOH waveguide using a cylindrical lens. Pump power is adjusted by sending the linearly polarized light from the pump laser through a half-wave plate and a polarizing beam splitter. Fibre 1 (cleaved SMF illuminating a GC) is used only to facilitate coarse alignment of Fibre 2 (lensed SMF) by using 1,550 nm light. (**b**) Emission from the SOH laser is collected by the lensed fibre (Fibre 2), which is connected to different detector setups by an optical switch. The upper path is used for high-sensitivity detection. It contains a monochromator and a slow but highly sensitive photodetector to record weak fluorescence. The sensitive PD has a low bandwidth, and a consecutive electrical low-pass filter is used to further suppress noise. The lower ‘fast-detection' path is used for time- and polarization-resolved measurements. It is equipped with fast PDs. Residual pump power is blocked by an optical long-pass filter.

The experimental setup is depicted in Fig. 5. The SOH devices are pumped from top by a pulsed laser at a wavelength of 1,064 nm with a duty cycle of approximately *p*_*t*_=1.23 × 10^−8^. The FWHM of the pump pulse amounts to 0.9 ns, the repetition frequency is 13.7 Hz. The incident pump power is controlled by adjusting the angle of a half-wave plate in front of a polarizing beam splitter. The pump light is polarized in a direction perpendicular to the waveguide axis and focused on the waveguide under test using a cylindrical lens, see Fig. 5a.

To measure emission from the SOH device, a lensed SMF is placed near the facet, denoted as ‘Fibre 2' in Fig. 5a. The fibre collects the emitted light with an estimated coupling loss of ∼5±1 dB. By coupling an auxiliary light beam at 1,550 nm through the on-chip GC to the SOH waveguide using a second fibre (Fibre 1), we can facilitate the alignment of the lensed Fibre 2 with respect to the waveguide facet. Polarization-maintaining fibres are used throughout the setup, and Fibre 2 is aligned such that the quasi-TE and quasi-TM emission of the SOH laser is coupled to the slow and the fast axis of the PM fibre, respectively. To characterize the laser emission, we use two different detection paths in our setup: A ‘high-sensitivity detection' path, corresponding to the upper part in Fig. 5b, and a ‘fast-detection' path, represented by the lower part in Fig. 5b.

The high-sensitivity path allows to measure input–output power characteristics and spectral properties of the laser emission. To this end, we use a monochromator and a highly sensitive photodetector with a large dynamic range, followed by an electrical low-pass filter for noise reduction and a standard oscilloscope, see Fig. 5b. The oscilloscope is triggered by the emission of the pump laser and averages over 16 subsequent pulses. Due to the electrical low-pass filter and the bandwidth limitations of both the photodetector and the oscilloscope, the recorded electrical pulse is strongly widened compared with its optical counterpart. However, the peak of the recorded electrical pulse still remains proportional to the received optical power. This setup allows measuring the wavelength-resolved emission spectrum. For high output powers, an attenuator (not shown) was inserted in front of the photodiode.

Time-resolved measurements are made with the fast-detection path. An optical long-pass filter blocks spurious pump light that might be scattered into the lensed fibre, and a polarization beam splitter is used to separate the two polarization states for individual detection. Light pulses with a duration in the (sub-)ns-range are detected with fast photodiodes (NewFocus 25-GHz model 1434, NewFocus 45 GHz model 1014). A high-speed oscilloscope (Tektronix DPO 70804B, 8 GHz bandwidth, 25 GSa s^−1^) is used to record time-resolved traces. The traces displayed in Fig. 4c have been obtained by averaging over 16 subsequent pulses. We find an average pump pulse duration of 0.9 ns FWHM with a standard error of 0.13 ns (15%). The durations of the emitted SOH laser pulses are shorter than that of the pump pulse. For quasi-TE polarization, the mean FWHM duration amounts to 0.6 ns with a standard error of ±0.06 ns (10%).

### Estimation of emission power levels

For high output powers above the lasing threshold, the peak power levels in the output fibre were measured using the fast-detection path of the setup depicted in Fig. 5, taking into account the responsivity of the fast photodiode and the optical and electrical losses of the various components. To obtain a lower boundary for the on-chip power levels, we assume that the total fibre–chip coupling losses are as low as 5 dB (factor 3.2). This value was estimated from reference measurements at 1,550 nm; the actual losses at 1,310 nm may be slightly higher. The coupling factor also includes losses of 6% due to reflection from the waveguide facet. A measured SOH laser peak power of 365 mW in the SMF hence corresponds to a laser peak power of at least 365 mW × 3.2 × 0.94=1.1 W which is coupled out from the waveguide facet and which could be used in an on-chip device that is connected to the SOH laser. To estimate the stochastic variations of the measured emission power, the high-speed detection path depicted in Fig. 5 is used. We record subsequent emission pulses from an SOH slot waveguide similar to the one depcited in Fig. 4a, pumped at powers of *P*_p1_=5 mW and *P*_p2_=15 mW, both of which are well above the threshold pump power of *P*_p,th_=2 mW. At *P*_p1_=5 mW, we find relative standard errors of approximately ±5%, and at *P*_p2_=15 mW, the relative standard error amounts to ±10%. For a conservative estimate, we assume that the relative standard error of the emitted power is ±10% for all pump powers of *P*_p1_=5 mW or more. Below pump powers of *P*_p1_=5 mW, we further assume a constant absolute error which corresponds to the ±10% relative standard error at *P*_p1_=5 mW. The range of negative powers is discarded, leading to the grey-shaded areas in Fig. 4b The grey-shaded areas in Fig. 2c,d were constructed in a similar way: For pump powers above *P*_p1_=5 mW, we assume a ±10% relative error, whereas for pump powers below 5 mW, we use a constant absolute error which corresponds to the ±10% relative standard error at *P*_p1_=5 mW. These uncertainty ranges are a coarse, but conservative estimate, which can only give a rough impression of the uncertainties of the measurement data.

For spectrally resolved measurements or for small power levels below the laser threshold, we use the high-sensitivity detection path of our setup. The peak power levels of the deformed pulses in the high-sensitivity path are calibrated by comparison with the corresponding peaks of the true pulse shapes in the fast-detection path using medium power levels that can reliably be detected in both paths.

### Estimation of pump power levels

While the total average pump power is directly accessible by measurement, the absorbed peak pump power needs to be estimated based on further assumptions. The elliptical Gaussian pump spot features a major axis of 8 mm and a minor axis of 0.3 mm, both defined by the FWHM of the intensity on the chip surface. This is much larger than the active area of the SOH waveguide, defined by the region in which pumped dye molecules interact with the lasing waveguide mode. Considering the example of the device depicted in Fig. 4, the length *l*_act, region_=3.8 mm of the active region is defined by the length of the slot waveguide section, and the width is estimated to the TE mode field diameter MFD_x_=0.77 μm in the lateral direction. The fraction of light that overlaps with the active zone is estimated by integrating the two-dimensional Gaussian distribution over the rectangle of MFD_x_ and waveguide length in the (*x*, *z*)-plane. This integral amounts to *p*_xz_=0.0027. To estimate the fraction *p*_y_ of pump light absorbed in the active cladding, we need to determine the corresponding absorption coefficient. From a direct transmission measurement using a 1.1-μm-thick IR26 dye-doped polymer layer on glass with the same dye concentration as the cladding material, the absorption cross section of the dispersed dye molecules is found to be *σ*_p_=1.7 × 10^−16^ cm^2^. This is in fair agreement with the value *σ*_p_=5 × 10^−16^ cm^2^ measured in a solution of the dye in 1, 2-dichloroethane^43^. The thickness of the cladding *h*_clad_=(500±50) nm has been measured using a profilometer. Using *σ*_p_=1.7 × 10^−16^ cm^2^ and a dye molecule concentration of *n*=10^19^ cm^3^, a value of *p*_y_=1−exp(−*σ*_p_*Nh*_clad_)=0.08 is found. The dye molecule number density *N* is derived from the measured mass ratio before mixing the PMMA matrix with the IR26 dyes. The total percentage of pump light absorbed in the active region is therefore *p*_xyz_=*p*_xz_ × *p*_y_=0.022%. Using the measured pump pulse shape and the duty cycle, we find a ratio of average pump power to peak pump power of *p*_avg/peak_=1.23 × 10^−8^, which leads to a ratio of average incident pump power to ‘absorbed' peak pump power of *p*=*p*_avg/peak_/*p*_xyz_=5.6 × 10^−5^. This ratio is used to relate the top and the bottom power scales in Fig. 4b. Consequently, the average incident threshold pump power of 1.8 mW leads to an estimate of the absorbed peak pump power of 32 W. The same method was used to relate the top and bottom power scales in Fig. 2c,d; the corresponding ratios of average incident power to absorbed peak power are listed in Supplementary Table 1. To estimate the variation of the measured pump power, a fraction of the pump pulse is coupled to a fibre and fed to a high-speed photodiode. From the measurements we find that the standard error of the peak pump power is ∼14%.

### Consistence of resonator characteristics and threshold pump power

The measured threshold pump powers of the SOH lasers are in reasonable agreement with the losses of the cavities. This is demonstrated by analysing the round-trip losses of a Fabry–Perot resonator with two cleaved facets as used in Fig. 4, and by relating them to the material gain of the active cladding.

The resonator round-trip losses are estimated by measuring the Fabry–Perot fringes in the transmission spectrum of the resonator and by evaluating the fringe contrast, see Supplementary Note 3 for a more detailed discussion. For TE polarization, we find a contrast ratio *C* of ∼0.5 dB between the transmission maxima and the adjacent minima, see Supplementary Fig. 4. According to Supplementary Note 3, this corresponds to a total round-trip loss of 10 log_10_(*a*^2^*R*^2^)=30.8 dB, where *R* denotes the power reflection factor at each facet and where *a* is the single-pass power transmission factor in the 3.8-mm long waveguide. This result is in good agreement with a bottom-up consideration: we use a finite-element solver^44^ to calculate the back-reflection *R* from the cleaved facet of an SOH waveguide, leading to a value of 6% (−12.2 dB), see Supplementary Table 1. Given the resonator length of *l*=3.8 mm and the total round-trip loss of 30.8 dB, we hence estimate a propagation loss of ∼0.9 dB mm^−1^ for the slot waveguide. This is in accordance with typically measured propagation losses of slot waveguides^45^ which are of the order of 1 dB mm^−1^.

At threshold, the round-trip losses of the resonator must be compensated by the round-trip amplification. For TE polarization, this requires a waveguide gain Γ_clad,TE_ *g*=−log*(aR)/l* corresponding to 4.1 dB mm^−1^, where Γ_clad,TE_=0.78 denotes the field interaction factor of the guided mode with the active cladding, see Supplementary Note 1 for more details. Laser emission in the dye cladding is governed by a transition that has a radiative lifetime^43^ of the order of 14 ns and a fluorescence quantum efficiency *φ* ranging from 0.02 to 0.1%, see (refs 43, 46). The effective lifetime of the excited state hence amounts to *φτ*≈3–14 ps—much shorter than the durations of the pump and the emission pulses. For estimating the pump intensity *I*_thresh_ at threshold, we may hence use steady-state approximations of the rate equations as described in detail in ref. 47 and Supplementary Note 3. This results in the relation


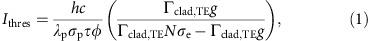


where *λ*_p_=1,064 nm is the pump wavelength, *σ*_p_=1.7 × 10^−16^ cm^2^ denotes the measured absorption cross section at this wavelength, *N* denotes the volume density of dye molecules, *τ*=14.4 ns is the radiative lifetime, *σ*_e_=0.5 × 10^−16^ cm^2^ is the emission cross-section^43^, and *φ* is the fluorescence quantum efficiency with typical values ranging from 0.02 to 0.1%, see (refs 43, 46), as specified for a liquid solution of the dye molecules.

When applied to the TE emission of the device depicted in Fig. 4, equation (1) leads to theoretically estimated threshold peak pump intensities ranging from 1.9 to 9.5 mW cm^−2^. This is in reasonable with agreement our experimental estimation of the threshold peak pump intensity of 13.7 mW cm^−2^. This estimation is based on the launched average threshold pump power of ∼1.8 mW, the overlap *p*_*xz*_=0.0027 of the active area with the Gaussian pump spot in the *x*, *z*-plane, the pump duty cycle of approximately *p*_t_=1.23 × 10^−8^, and the area of the active zone having a length of *l*=3.8 mm and a width of MFD_x_=0.77 μm.

The deviations between the measured and the predicted the peak pump intensity is attributed to large uncertainties of the quantum efficiency *φ*. Previously published figures range from 0.02 to 0.1% and were measured in liquid dye solutions, see refs 43, 46, whereas we use the dyes in a solid polymer matrix. The measured value of 13.7 mW cm^−2^ for the peak pump intensity can be reproduced by equation (1) when assuming a quantum efficiency of *φ*=0.014%—which is comparable to the values obtained for liquid dye solutions. In addition, it turns out that FCA may additionally increase the cavity losses, see the section ‘Optically induced losses and dynamical behaviour' of the Methods for more details. This would explain the fact that the experimentally measured threshold is slightly larger than the theoretically predicted value and lead to quantum efficiencies that are even closer to previously published values.

For TM polarization, the measured contrast of the Fabry–Perot fringes is comparable to that for TE polarization. Both polarizations hence experience similar cavity losses. Figure 4b shows a slightly increased threshold pump power of the TM compared with the TE mode—this is attributed to a reduced field interaction factor of Γ_clad,TM_=0.42 in the cladding compared with Γ_clad,TE_=0.78. Moreover, the TM mode experiences higher FCA than the TE mode due to a stronger field interaction with the silicon waveguide core, see the section ‘Optically induced losses and dynamical behaviour' below.

### Optically induced losses and dynamical behaviour

The dynamical behaviour of the laser emission is depicted in Fig. 4c. In this figure, the relative timing of the pump pulse and the emission pulses is subject to uncertainties: The various traces for the pump pulse, the TE emission and the TM emission were measured by an oscilloscope and a photodetector connected to the chip by standard SMF (G.652). For measuring the TE and the TM emission pulse, light was collected from the same fibre facet, and we may assume that both pulse trains experience the same propagation delay in the fibre. This is different for the pump—for measuring the pump pulse trace, we first had to remove the long-pass filter that was used to suppress residual pump light before it reaches the detector. We then moved the lensed fibre (Fibre 2 in Fig. 5) laterally to collect a small portion of 1,064 nm pump light scattered from the surface of the chip. The group delay of the pump pulses from the fibre tip to the detector is slightly different than that of the emission pulses since the optical setup had to be changed slightly and since the optical fibre is operated below its single-mode cutoff wavelength of 1,260 nm. This leads to higher-order mode propagation and hence to further uncertainties of the group delay. The overall uncertainty in relative timing between the pump and the emission pulses is estimated to be ±100 ps.

We also investigated the dynamics of intra-cavity losses at the emission wavelength of 1,310 nm. The influence of two-photon absorption (TPA) of the emitted light and TPA-induced FCA can be neglected, see Supplementary Note 4. As the only relevant loss mechanism, we identify FCA induced by direct absorption of 1,064 nm pump light in the silicon waveguide core: during the pump pulse, free carriers accumulate within the core of the silicon waveguide, thereby leading to absorption and considerably increasing the optical losses of the resonator also at the emission wavelength. For a rough quantitative estimate, we assume a linear absorption coefficient of 10 cm^−1^ for the 1,064 nm pump light in the silicon waveguide core^48^. During pumping, photons absorbed in the waveguide create pairs of free carriers with an effective lifetime^45^ of the order of 1 ns. Similarly to the considerations made for the active region of the SOH laser, the fraction of pump light that overlaps with the silicon waveguide is estimated to be *p*_xz, Si_=0.0011, and the fraction of pump light absorbed in the 220 nm high silicon waveguide core is estimated to *p*_y, Si_=0.00022. Using these values, the free-carrier density would reach 6.6 × 10^17^ cm^−3^ for an average pump power of 1.8 mW, corresponding to the threshold of the laser depicted in Fig. 4. For this carrier density, an empirical model^48^ allows us to roughly estimate an upper limit of the FCA-related propagation loss of ∼5 dB mm^−1^ in the silicon core at the end of the pump pulse. Additional losses of this magnitude may significantly reduce the quality of the optical resonator during pumping and lead to an increased threshold. This is consistent with the observation that the experimentally measured threshold is slightly larger than the theoretically predicted value. We expect that in future devices, FCA can be mitigated by pumping at infrared wavelengths, which are not absorbed in the SOI waveguide core, or by using reverse-biased p-i-n structures that remove free carriers from the silicon core of the waveguides^7^. That would allow to considerably reduce threshold pump powers and to increase the slope efficiencies of the devices.

### Summary of resonator and laser emission characteristics

For the quantitative estimations in this paper, various waveguide and resonator parameters are used. These parameters are summarized in Supplementary Table 1 along with threshold and emission power levels of the respective devices. The values are obtained either from experiments or from numerical simulations, for example, for the case of the field interaction factor, effective area^49^ and mode field diameter. The underlying mathematical relations are given in Supplementary Note 1.

## Additional information

**How to cite this article:** Korn, D. *et al.* Lasing in silicon–organic hybrid waveguides. *Nat. Commun.* 7:10864 doi: 10.1038/ncomms10864 (2016).

## Supplementary Material

Supplementary Information Supplementary Figures 1-4, Supplementary Table 1, Supplementary Notes 1-4 and Supplementary References.
