# Supplementary material for: Lasing in silicon–organic hybrid waveguides
Source: Nat Commun. 2016 Mar 7;7:10864. doi: 10.1038/ncomms10864 (PMC4786640; doi:10.1038/ncomms10864)
Supplement: Supplementary Information — Supplementary Figures 1-4, Supplementary Table 1, Supplementary Notes 1-4 and Supplementary References. [file ncomms10864-s1.pdf]

Lasing in Silicon-Organic Hybrid Waveguides
# - Supplementary Information -

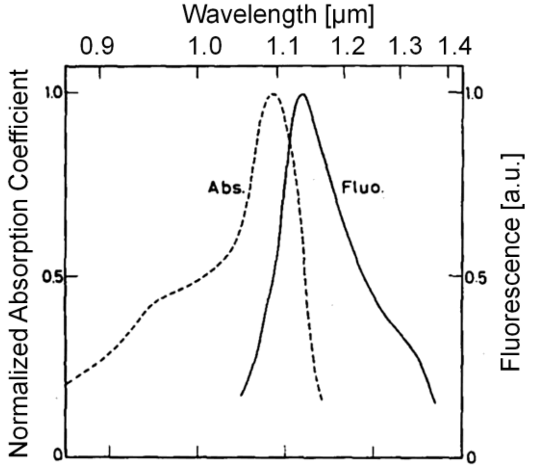

Supplementary Figure 1. Absorption and fluorescence spectra of the organic dye IR-26[1], dissolved in 1,2-dichloroethane. The exact shapes of these spectra depend on the host material. Due to self-absorption in the cladding of the silicon waveguides, the emission peak is shifted towards 1300 nm compared to the depicted fluorescence spectrum in solution[2]. In our experiment, the material is pumped at a wavelength of 1.064 µm, thus close to the wavelength of maximum absorption of 1080 nm. [Reprinted from *Optics Communications* **36**, 149–152, Kranitzky *et al.*, "A new infrared laser dye of superior photostability tunable to 1.24 µm with picosecond excitation.", Fig. 2, (1981) with permission from Elsevier. Copyright.com license number: 3785270713354.]

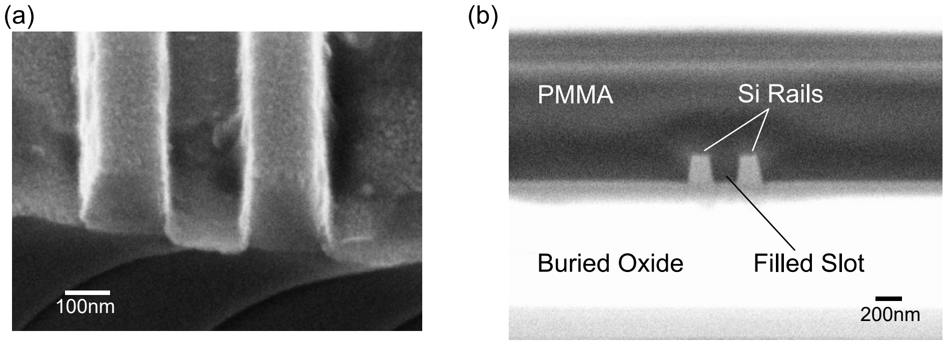

Supplementary Figure 2. Scanning electron microscope (SEM) images of fabricated slot-waveguide samples. The structures are nominally identical to the one used for the SOH laser in Fig. 4 of the main paper. (a) Cleaved facet of an SOI slot waveguide after removing the PMMA cladding. (b) Cross-sectional view of a SOH slot waveguide coated by an undoped PMMA cladding. The figure was obtained by focused ion beam (FIB) milling and scanning electron microscopy. The cladding was deposited by spin coating and fills the slot completely without forming any voids.

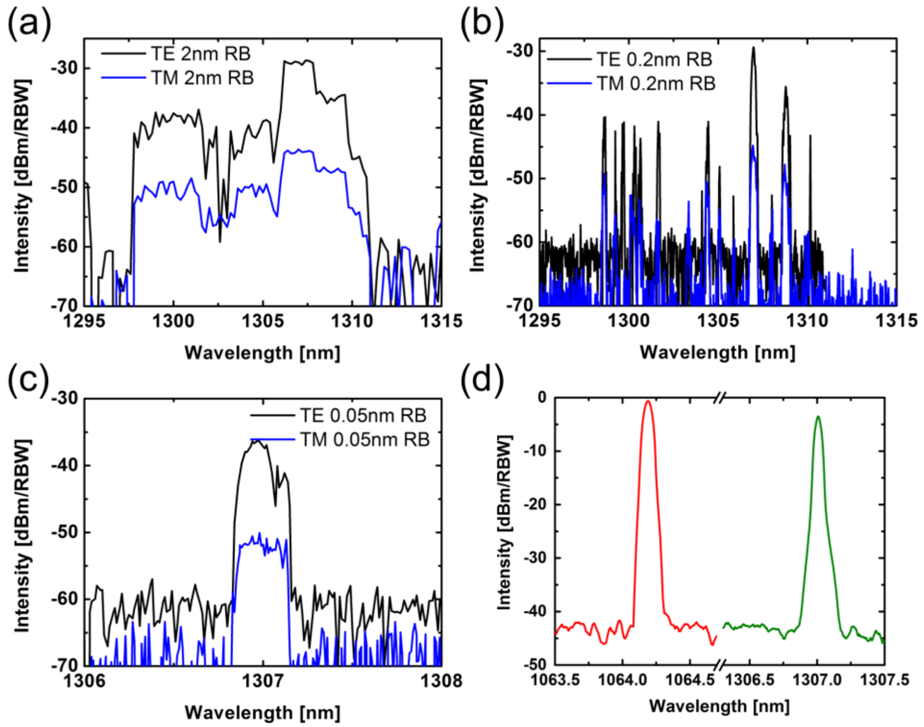

Supplementary Figure 3. High-resolution laser emission spectra in TE and TM polarization derived from the slot-waveguide laser that was also used in Fig. 2 of the main paper. Fiber-chip coupling has been re-optimized for each polarization-resolved measurement. We use an optical spectrum analyzer operated at a hold time of 170 ms for each spectral point. As a consequence, at least two pulses at a repetition rate of 13.7 Hz are recorded at each wavelength. **(a)** Overview spectrum at a resolution bandwidth (RBW) of 2 nm. **(b)** Scan with 0.2 nm resolution revealing a multitude of underlying narrowband spectral lines, which we attribute to longitudinal cavity modes. Repeated scans show peaks at positions that are indistinguishable from scan to scan within the measurement accuracy. We conclude that the same set of longitudinal modes starts lasing simultaneously in each pulse and contributes to the overall output power. A single spectral line at approximately 1307 nm dominates the emission spectrum and contains the majority of the optical power. **(c)** Scan with 0.05 nm resolution. A single emission peak of one longitudinal mode exhibits a linewidth of around 0.2 nm. We attribute this to chirp-induced spectral broadening due intra-cavity free-carrier dynamics induced by absorption of 1064 nm pump light in the silicon waveguide cores, see Supplementary Note 2. **(d)** Spectra of the pump 1064 nm pump laser and of a 1307 nm external-cavity continuous-wave laser. In both cases, the width of the measured spectrum is solely determined by the resolution of the spectrometer. These measurements have been taken as control experiments to verify that the emission linewidth of around 0.2 nm visible in (c) is indeed linked to the emission of the laser and not caused by the resolution of the spectrometer.

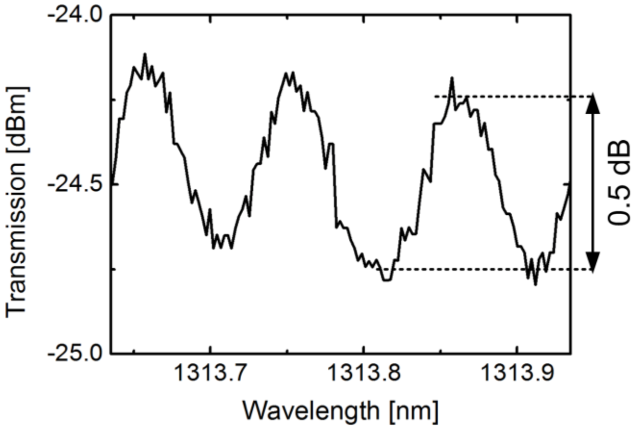

Supplementary Figure 4. Close-up of a transmission spectrum of a slot waveguide with cleaved facets, measured at TE polarization using a tunable external-cavity laser. The free spectral range corresponds to a Fabry-Perot cavity of 3.75 mm with a group refractive index of 2.1. The contrast of the fringes of 0.5 dB corresponds to a roundtrip loss of 30.8 dB.

Property	Fig. 2(a) Strip waveguide	Fig. 2(b) Slot waveguide	Fig. 4 Slot waveguide
Height	h_{WG} = 220 nm, $h_{clad} \approx$ (500±50) nm		
Width	$w_{strip} \approx$ 210 nm	$w_{rail} \approx$ 180 nm<br>$w_{slot} \approx$ 215 nm	w_{rail} = (160±15) nm<br>w_{slot} = (180±15) nm
Length	$l_{complete}$ = 4.8 mm<br>$l_{act.\ region}$ = 4.3 mm	$l_{complete}$ = 4.8 mm<br>$l_{act.\ region}$ = 3.8 mm	$l_{complete}$ = 3.8 mm<br>$l_{act.\ region}$ = 3.8 mm
Optical feedback	grating coupler + cleaved facet	grating coupler + cleaved facet	two cleaved facets
Effective refractive index (simulation)	n_{eff} = 1.71	n_{eff} = 1.67	n_{eff} = 1.61
Facet power reflectivity (simulation) for TE-mode	8%<br>-10.8 dB	6%<br>-12.2 dB	6%<br>-12.2 dB
Facet power reflectivity (simulation) for TM-mode	5%<br>-12.6 dB	5%<br>-13.0 dB	4%<br>-13.6 dB
Propagation loss at 1310 nm	(12±5) dB·cm^{-1}	(9±2) dB·cm^{-1}	
Field interaction factors with active cladding (clad) and silicon (Si) core for TE and TM polarization (simulation)	$\Gamma_{clad,\ TE} \approx 0.64$<br>$\Gamma_{clad,\ TM} \approx 0.42$<br>$\Gamma_{Si,\ TE} \approx 0.29$<br>$\Gamma_{Si,\ TM} \approx 0.34$	$\Gamma_{clad,\ TE} \approx 0.76$<br>$\Gamma_{clad,\ TM} \approx 0.54$<br>$\Gamma_{Si,\ TE} \approx 0.17$<br>$\Gamma_{Si,\ TM} \approx 0.29$	$\Gamma_{clad,\ TE} \approx 0.78$<br>$\Gamma_{clad,\ TM} \approx 0.42$<br>$\Gamma_{Si,\ TE} \approx 0.12$<br>$\Gamma_{Si,\ TM} \approx 0.26$
Effective mode cross-section for third-order nonlinearities, (simulation for TE-mode)	$A_{eff,\ clad}$ = 0.15 µm^2<br>$A_{eff,\ Si}$ = 0.15 µm^2	$A_{eff,\ cladd}$ = 0.20 µm^2<br>$A_{eff,\ Si}$ = 0.89 µm^2	$A_{eff,\ cladd}$ = 0.19 µm^2<br>$A_{eff,\ Si}$ = 1.56 µm^2
Mode field diameter in *x*-direction (simulation for TE-mode)	MFD_x = 0.66 µm	MFD_x = 0.79 µm	MFD_x = 0.77 µm
Ratio of average incident power to absorbed peak power	$p_{avg/peak} / p_{xyz} = 6.0 \times 10^{-5}$	$p_{avg/peak} / p_{xyz} = 5.5 \times 10^{-5}$	$p_{avg/peak} / p_{xyz} = 5.6 \times 10^{-5}$
**SOH laser performance**			
Launched average threshold pump power	2.3 mW	1.3 mW	1.8 mW
Absorbed peak threshold power	38 W	24 W	32 W
Laser emission peak power, at a wavelength of 1310 nm	150 mW (in SMF)	270 mW (in SMF)	365 mW (in SMF)<br>1.1 W (output facet)<br>1.2 W (in resonator)

Supplementary Table 1. Waveguide properties, resonator parameters, and laser performance overview. All parameters refer to quasi-TE modes at a wavelength of 1310 nm, unless stated otherwise. The slot waveguides turn out to have a larger confinement of light to the cladding than the strip waveguides. This leads to a stronger interaction of the guided mode with the active cladding. The resonators in Fig. 2 of the main paper include wide silicon waveguides sections. These sections consist of the grating couplers (GC) and access waveguides or transitions, in which the laser light is tightly confined to the Si waveguide core and hence only a very minor part interacts with the active cladding. Consequently, only the narrow strip section or the slot section contribute to lasing and are regarded as part of the active region. Therefore the device lengths $l_{complete}$ and the lengths $l_{act.\ region}$ of the active region are stated separately.

Supplementary Note 1: Waveguide parameters

For the quantitative estimations in the main paper, various waveguide parameters are used. These parameters are summarized in Supplementary Table 1 along with threshold and emission power levels of the respective devices. The values are obtained either from experiments or from numerical simulations, e.g., for the case of the field interaction factor, effective area[3] and mode field diameter. The underlying mathematical relations are given in the following.

The observed output power of the slot-waveguide laser, Fig. 2(d) of the main paper, is larger than the output power of the strip waveguide, Fig. 2(c), and the lasing threshold is also lower for the slot waveguide. To understand this behavior, not only the resonator but also the available gain has to be considered. The available gain and dynamic loss depend on the distribution of light in the waveguide cross-section. The overlap of the guided light with the active organic cladding can be quantified by means of the field interaction factor given by[4]

$$\Gamma = \frac{\int\limits_{\text{reg}} \frac{n_{\text{reg}}}{Z_0} \left|\boldsymbol{E}(x,y)\right|^2 \,\mathrm{d}x\,\mathrm{d}y}{\int\limits_{\text{all}} \Re\left\{\boldsymbol{E}(x,y)\times\boldsymbol{H}^*(x,y)\right\}\cdot\boldsymbol{e}_z \mathrm{d}x\,\mathrm{d}y}. \tag{1}$$

In this relation, the refractive index of the active polymer cladding (reg, integration region of the numerator integral) is denoted as n_{reg}, Z_0 is the vacuum wave impedance, $\mathbf{e}_z$ the unit vector in z-direction, and $\boldsymbol{E}(x,y)$ and $\boldsymbol{H}(x,y)$ denote the electric and magnetic mode fields in the transverse (x,y)-plane. The integration domain of the numerator integral corresponds to the active cladding of the waveguide, whereas the denominator extends over the entire waveguide cross section. When calculating the field interaction with the active cladding region according Eq. (1), we find that the slot waveguide of Fig. 2(b) exhibits an interaction factor $\Gamma_{\text{clad, TE, slot}} \approx 0.76$ that is slightly larger than the value obtained for the strip waveguide $\Gamma_{\text{clad, TE, strip}} \approx 0.64$ in Fig. 2(a), see Supplementary Table 1 for a summary of waveguide parameters.

For nonlinear optical effects such as two-photon absorption, the concentration of light within a certain part of the waveguide, e.g., the silicon waveguide core, must be quantified. This is usually done by means of the effective area of third-order nonlinear interaction. The effective area for interaction with a certain cross-sectional region (reg) of the waveguide is given by[3]

$$A_{\mathrm{eff}} = \frac{\left(\int_{\mathrm{all}} \Re\left\{ \boldsymbol{E}(x,y) \times \boldsymbol{H}^{*}(x,y) \right\} \cdot \boldsymbol{e}_z \mathrm{d}x\mathrm{d}y \right)^2}{\int_{\mathrm{reg}} \frac{n_{\mathrm{reg}}^2}{Z_0^2} \left| \boldsymbol{E}\ (x,y) \right|^4 \mathrm{d}x\mathrm{d}y}, \tag{2}$$

where $\boldsymbol{E}(x,y)$ and $\boldsymbol{H}(x,y)$ denote again the electric and magnetic mode fields in the transverse (x,y)-plane. For the strip waveguide, we find that the effective area of third-order nonlinear interaction in the silicon core amounts to $A_{\mathrm{eff,\,Si,\,strip}} = 0.15\ \mu\mathrm{m}^2$ and is much smaller than its slot-waveguide counterpart, $A_{\mathrm{eff,\,Si,\,slot}} = 0.89\ \mu\mathrm{m}^2$. Hence, for the same power of guided light, any non-linear effects in the waveguide core such as two-photon absorption will be much stronger in the strip waveguide than in the slot waveguide as discussed in Supplementary Note 4.

For describing the spatial extent of the gain region of an active SOH waveguide, we use the second-moment width $\mathrm{MFD_x} = \mathrm{D}4\sigma$, i.e., four times the second central moment along the x-direction,

$$\mathrm{MFD}_x = 4\sqrt{\frac{\int_{\mathrm{reg}} \frac{n_{\mathrm{reg}}}{Z_0} \left| \boldsymbol{E}_{\mathrm{t}}(x,y) \right|^2 (x-\bar{x})^2 \,\mathrm{d}x\mathrm{d}y}{\int_{\mathrm{all}} \Re\left\{ \boldsymbol{E}_{\mathrm{t}}(x,y) \times \boldsymbol{H}_{\mathrm{t}}^{*}(x,y) \right\} \cdot \boldsymbol{e}_z \mathrm{d}x\mathrm{d}y}}. \tag{3}$$

Note that the first moment $\bar{x}$ is zero, because the waveguides and the magnitude of the resulting electric mode fields are symmetric with respect to the (y,z)-plane. The mode field diameter is $\mathrm{MFD_x} = 0.66\ \mu\mathrm{m}$ ($\mathrm{MFD_x} = 0.79\ \mu\mathrm{m}$) for the strip (slot) waveguide in Fig. 2, see Supplementary Table 1. Hence we may expect the number of excited dye molecules available for stimulated emission to be slightly larger for the slot waveguide than for the strip waveguide.

Supplementary Note 2: High-resolution spectra

To explain the comparatively large bandwidth of laser emission in Fig. 2, we have investigated the emission spectrum at higher resolutions of 2 nm, 0.2 nm, and 0.05 nm, see Supplementary Figure 3 (a) to (c). We investigated both TE and TM polarization. The hold time of the optical spectrum analyzer at each measurement point amounts to 170 ms, and is chosen such that at least two emission pulses at a repetition rate of 13.7 Hz are recorded. We find that the spectra consist of a multitude of narrowband spectral lines, which we attribute to different longitudinal modes of the cavity that exhibit laser emission simultaneously in each cycle. The positions of these emission peaks are reproducible when measuring the spectrum repeatedly. A single spectral line at approximately 1307 nm dominates the emission spectrum and contains the majority of the optical power. A zoom-in of this line is depicted in Supplementary Figure 3 (c), exhibiting a FWHM linewidth of 0.1 … 0.2 nm. This linewidth is much larger than the 0.003 nm FWHM expected for an unchirped pulse of 1 ns duration. We attribute the excess spectral bandwidth to a strong chirp, induced by free-carrier dynamics of the cavity which originate from absorption of 1064 nm pump light in the silicon waveguide cores and which lead to a strong change of the cavity refractive index during pulse emission. As a control experiment, we recorded the spectra of both the pump and of a 1307 nm external-cavity continuous-wave (CW) laser using a RBW of 0.05 nm, see Supplementary Figure 3 (d). The linewidth of both lasers is significantly smaller than the resolution bandwidth of the spectrometer, and hence the recorded spectra in Supplementary Figure 3 (d) reveal the line shape of the monochromator used in the spectrometer. For the 1307 nm CW laser, the recorded spectrum appears much narrower than the emission spectrum of the SOH laser in Supplementary Figure 3 (c). This confirms that the spectral linewidth of the SOH laser obtained from Supplementary Figure 3 (c) is indeed linked to the emission of the laser and not caused by the resolution of the spectrometer. The fact that both the TE and the TM emission exhibit spectral narrowing above threshold is another strong indication for laser emission into both modes. A striking similarity of the TE and TM spectra is observed at highest resolution, Supplementary Figure 3 (c). We consider this as an indication that the TE and TM modes are coupled: TE starts lasing at lower pump powers, and a small portion of the TE light will couple to the TM mode and act as a seed for lasing.

Supplementary Note 3: Resonator loss and pump threshold

Estimation of resonator losses

For a Fabry-Perot resonator, the phase shift δ accumulated during one round trip can be calculated according to

$$\delta = 2 n_e l \frac{2\pi}{\lambda}, \tag{4}$$

with n_e denoting the effective refractive index in the waveguide of length l and λ being the vacuum wavelength. The transmission through the resonator depends on the incident wavelength and the resonator parameters. Let R denote the facet power reflectivity and a the single-pass power transmission factor. The wavelength-dependent power transmission T of the Fabry-Perot resonator can then be written as

$$T = \frac{\left(1 - aR\right)^2}{1 + a^2 R^2 - 2aR\cos\delta}. \tag{5}$$

From this relation, we can derive an expression for the fringe contrast *C,* i.e., the ratio of the power transmission maxima and the adjacent minima, similar as for the Hakki Paoli Method[5]

$$C = \frac{(1 + aR)^2}{(1 - aR)^2}. \tag{6}$$

The fringe contrast C is obtained from a high-resolution transmission spectrum of the resonator, see, e.g., Supplementary Fig. 4. Solving Eq. (6) for the single-pass power transmission factor aR of the Fabry Perot resonator, we obtain 10 log(aR) = -15.4 dB for the slot waveguide presented in Fig. 4. This corresponds to a 30.8 dB round-trip loss. We can also determine the coefficient of finesse F of the resonator using the relation

$$F = \frac{4aR}{\left(1 - aR\right)^2} \tag{7}$$

For the slot waveguide presented in Fig. 4, the coefficient of finesse is 0.12.

Calculation of pump threshold

Assuming a quasi four-level system and neglecting the triplet states, the simplified rate equation can be written as[6]

$$\frac{\mathrm{d}N_1}{\mathrm{d}t} = N_0 \left(\sigma_\mathrm{p} \frac{I_\mathrm{p}}{hc/\lambda_\mathrm{p}} + \sigma_a \frac{I_\mathrm{e}}{hc/\lambda_\mathrm{e}} \right) - N_1 \left(\sigma_\mathrm{e} \frac{I_\mathrm{e}}{hc/\lambda_\mathrm{e}} + \frac{1}{\tau\phi} \right) \tag{8}$$

In this equation, the volume density of dye molecules in the ground state (level 0) is denoted by N_0, whereas N_1 denotes the volume density of dye molecules in the excited level (level 1). The total density of dye molecules is $N = N_1 + N_0$. The wavelength and the intensity of the emitted light are given by λ_e and I_e, respectively, and λ_p and I_p denote the corresponding quantities for the pump light. Plank's constant is denoted as h and the velocity of light as c. The quantity τ is the decay time associated with radiative transitions from the excited state to the ground state, and ϕ is the fluorescence quantum yield. The emission cross section is denoted as σ_e, the absorption cross section at the emission frequency is called σ_a and the absorption at the pump frequency is σ_p.

For 1 wt% dye in polymer, the total volume density of dye molecules amounts to $N = 1 \times 10^{19}\ \mathrm{cm}^{-3}$. For the excited state transition lifetime and the emission cross section at 1310 nm we use values from literature[7], $\tau = 14.4$ ns and $\sigma_e = 0.5 \times 10^{-16}\ \mathrm{cm}^2$. Reabsorption at the emission wavelength can be neglected, $\sigma_a = 0$. The absorption cross section σ_p at the pump wavelength λ_p of 1064 nm was obtained from a transmission measurement of a dye-doped polymer film on a glass substrate and amounts to $\sigma_p = 1.7 \cdot 10^{-16}\ \mathrm{cm}^2$.

For $\phi \approx 0.1\%$, the decay of excited states is governed by the time constant $\tau\phi \approx 14\,\mathrm{ps}$. This is much shorter than the duration of the 0.9 ns pump pulse, and we may hence consider the steady-state behavior of Eq. (8) to estimate the threshold intensity. Assuming further that the emission intensity I_e is still zero at threshold, the density of excited state molecules is obtained from Eq. (8),

$$N_1 = \frac{N\sigma_p I_p \tau\phi}{\sigma_p I_p \tau\phi + hc/\lambda_p}\,. \tag{9}$$

At this density, the single-pass gain must compensate the single-pass losses in the resonator, thus $\Gamma_{\mathrm{clad}} N_1 \sigma_e = \Gamma_{\mathrm{clad}}\, g = -\log(aR)/l$, where Γ_{clad} denotes the field interaction factor with the active cladding as defined in Eq. (1) and listed in Supplementary Table 1. The threshold pump intensity is thus obtained to

$$I_{\mathrm{p,thres}} = \frac{hc}{\lambda_p}\frac{1}{\tau\phi\sigma_p}\left(\frac{-\log(aR)}{l\Gamma_{clad} N\sigma_e + \log(aR)}\right). \tag{10}$$

For the slot waveguide depicted in Fig. 4, we experimentally determine a launched peak pump threshold intensity of $I_{\mathrm{p,thresh}} = 13.7\ \mathrm{MW \cdot cm^{-2}}$. This estimation is based on the launched average threshold pump power of approximately 1.8 mW, the overlap $p_{xz} = 0.0027$ of the active area with

the Gaussian pump spot in the (*x,z*)-plane, the pump duty cycle of approximately $p_t = 1.23 \times 10^{-8}$, and the area of the active zone having a length of $l = 3.8$ mm and a width of $\mathrm{MFD_x} = 0.77$ µm. Using these parameters, the experimentally determined peak pump threshold intensity of $I_{\mathrm{p,thresh}} = 13.7\ \mathrm{MW{\cdot}cm^{-2}}$ can be reproduced by Eq. (10) if we assume a quantum efficiency of 0.014 %. This value is close to published quantum efficiencies ranging from 0.02% to 0.1%, see Refs. [8,9]. The remaining deviations are attributed to the fact that the references refer to a liquid solution of the dye molecules rather than to a solid polymer matrix as used in our experiments. Moreover, free-carrier absorption contributes additional cavity loss and leads to an increase of the pump threshold, see Section 6 of the Methods in the main paper.

Supplementary Note 4: Optically induced nonlinear losses

Losses for light propagating in silicon at the emission wavelength of $\lambda_{\mathrm{e}} = 1300\,\mathrm{nm}$ comprise linear waveguide losses, two-photon absorption (TPA), and absorption by free carriers that are generated as a result of TPA. The associated decay of intensity I along the propagation direction z can be approximated by a first-order differential equation[10],

$$\frac{dI(z)}{dz} = -\left(\alpha_{\mathrm{lin}} + \beta_{\mathrm{TPA}} I(z) + \alpha_{\mathrm{FCA}} I^2(z)\right) I(z)\,. \tag{11}$$

In this relation α_{lin} denotes the linear propagation loss in the SOI waveguide, $\beta_{\mathrm{TPA}} = 0.74\,\mathrm{cm}\cdot\mathrm{GW}^{-1}$ denotes the TPA coefficient of bulk silicon[11], and α_{FCA} is the coefficient of TPA-induced free-carrier absorption (FCA)[10],

$$\alpha_{\mathrm{FCA}} = 1.45\times 10^{-21}\,\mathrm{m}^2 \left(\frac{\lambda_{\mathrm{e}}}{1.55\,\mu\mathrm{m}}\right)^2 \frac{\beta_{\mathrm{TPA}}\tau_{\mathrm{eff,\,Si}}\lambda_{\mathrm{e}}}{2hc}\,, \tag{12}$$

where $\tau_{\mathrm{eff,\,Si}}$ denotes the effective free-carrier lifetime in the silicon waveguide core and amounts to $\tau_{\mathrm{eff,\,Si}} = 1\,\mathrm{ns}$ [12]. Note that this lifetime is much shorter than the carrier lifetime in bulk silicon due to increased recombination rates at the etched waveguide surfaces.

For an exemplary estimation of the influence of two-photon absorption of 1310 nm emission, we consider the slot waveguide of Fig. 4 and assume an emitted laser peak power of $P_{\mathrm{facet}} = 1.1\,\mathrm{W}$ just outside the facet. The actual power inside the laser resonator is even higher due to the 6 % power reflectivity of the facet and is estimated to be approximately 1.2 W. Only a part of the light is guided in the silicon waveguide core with an effective mode cross section of $A_{\mathrm{eff,\,Si}} = 1.56\,\mu\mathrm{m}^2$, see Supplementary Table 1. This leads to an intensity estimate of $I_{1310\,\mathrm{nm}} = 0.077\,\mathrm{GW}\cdot\mathrm{cm}^{-2}$, resulting in a TPA-induced loss contribution of 0.03 $\mathrm{dB}\cdot\mathrm{mm}^{-1}$ for this specific power level. Compared to linear losses in the waveguide of 0.9 $\mathrm{dB}\cdot\mathrm{mm}^{-1}$, TPA is thus negligible as a direct loss mechanism in our case. To obtain an upper boundary for the TPA-induced FCA, we assume an optical continuous-wave (cw) signal with an average power that corresponds to the 1.2 W peak power of our emission pulse. This leads to TPA-induced FCA coefficient of $\alpha_{\mathrm{FCA\ from\ TPA}} = 2.5\times 10^{-23}\,\mathrm{m}^3\mathrm{W}^{-2}$ and thus to a loss contribution of less than 0.06 $\mathrm{dB}\cdot\mathrm{mm}^{-1}$ at peak of the emitted laser pulse. The contribution of TPA-induced FCA to waveguide losses can hence also be safely neglected. In contrast to that, FCA induced by direct

absorption of the 1064 nm pump light turns out to be a relevant effect, see Section 6 of the Methods of the main paper for more details.

Supplementary References

1. Kranitzky, W., Kopainsky, B., Kaiser, W., Drexhage, K. H. & Reynolds, G. A. A new infrared laser dye of superior photostability tunable to 1.24 μm with picosecond excitation. *Optics Communications* **36,** 149–152 (1981).
2. Casalboni, M. *et al.* 1.3 μm light amplification in dye-doped hybrid sol-gel channel waveguides. *Applied Physics Letters* **83,** 416 (2003).
3. Koos, C., Jacome, L., Poulton, C., Leuthold, J. & Freude, W. Nonlinear silicon-on-insulator waveguides for all-optical signal processing. *Opt. Express* **15,** 5976 (2007).
4. Brosi, J.-M. *et al.* High-speed low-voltage electro-optic modulator with a polymer-infiltrated silicon photonic crystal waveguide. *Opt. Express* **16,** 4177–4191 (2008).
5. Hakki, B. W. & Paoli, T. L. Gain spectra in GaAs double−heterostructure injection lasers. *J. Appl. Phys.* **46,** 1299 (1975).
6. Shank, C. V. Physics of dye lasers. *Rev. Mod. Phys.* **47,** 649–657 (1975).
7. Benfey, D. P., Brown, D. C., Davis, S. J., Piper, L. G. & Foutter, R. F. Diode-pumped dye laser analysis and design. *Applied Optics* **31,** 7034 (1992).
8. Semonin, O. E. *et al.* Absolute Photoluminescence Quantum Yields of IR-26 Dye, PbS, and PbSe Quantum Dots. *The Journal of Physical Chemistry Letters* **1,** 2445–2450 (2010).
9. Benfey, D. P., Brown, D. C., Davis, S. J., Piper, L. G. & Foutter, R. F. Diode-pumped dye laser analysis and design. *Appl. Opt.* **31,** 7034–7041 (1992).
10. Turner-Foster, A. C. *et al.* Ultrashort free-carrier lifetime in low-loss silicon nanowaveguides. *Optics Express* **18,** 3582 (2010).
11. Dinu, M., Quochi, F. & Garcia, H. Third-order nonlinearities in silicon at telecom wavelengths. *Applied Physics Letters* **82,** 2954–2956 (2003).
12. Vallaitis, T. *et al.* Optical properties of highly nonlinear silicon-organic hybrid (SOH) waveguide geometries. *Opt. Express* **17,** 17357–17368 (2009).
